# Supplementary material for: A perceptual scaling approach to eyewitness identification
Source: Nat Commun. 2020 Jul 14;11:3380. doi: 10.1038/s41467-020-17194-5 (PMC7360747; doi:10.1038/s41467-020-17194-5)
Supplement: Supplementary file 2 — Reporting Summary [file 41467_2020_17194_MOESM2_ESM.pdf]

nature research

Corresponding author(s): Thomas D. Albright

Last updated by author(s): Mar 26, 2020

Reporting Summary

Nature Research wishes to improve the reproducibility of the work that we publish. This form provides structure for consistency and transparency in reporting. For further information on Nature Research policies, see Authors & Referees and the Editorial Policy Checklist.

Statistics

For all statistical analyses, confirm that the following items are present in the figure legend, table legend, main text, or Methods section.

n/a	Confirmed	
☐	☒	The exact sample size (*n*) for each experimental group/condition, given as a discrete number and unit of measurement
☐	☒	A statement on whether measurements were taken from distinct samples or whether the same sample was measured repeatedly
☐	☒	The statistical test(s) used AND whether they are one- or two-sided<br>*Only common tests should be described solely by name; describe more complex techniques in the Methods section.*
☐	☒	A description of all covariates tested
☐	☒	A description of any assumptions or corrections, such as tests of normality and adjustment for multiple comparisons
☐	☒	A full description of the statistical parameters including central tendency (e.g. means) or other basic estimates (e.g. regression coefficient) AND variation (e.g. standard deviation) or associated estimates of uncertainty (e.g. confidence intervals)
☐	☒	For null hypothesis testing, the test statistic (e.g. F, t, r) with confidence intervals, effect sizes, degrees of freedom and P value noted<br>*Give P values as exact values whenever suitable.*
☒	☐	For Bayesian analysis, information on the choice of priors and Markov chain Monte Carlo settings
☒	☐	For hierarchical and complex designs, identification of the appropriate level for tests and full reporting of outcomes
☒	☐	Estimates of effect sizes (e.g. Cohen's *d*, Pearson's *r*), indicating how they were calculated

Our web collection on statistics for biologists contains articles on many of the points above.

Software and code

Policy information about availability of computer code

Data collection	Data were collected using the software platform MATLAB, Release 2017b, The MathWorks, Inc., Natick, Massachusetts, United States.
Data analysis	Data were analyzed using the software platform MATLAB, Release 2017b, The MathWorks, Inc., Natick, Massachusetts, United States.

For manuscripts utilizing custom algorithms or software that are central to the research but not yet described in published literature, software must be made available to editors/reviewers. We strongly encourage code deposition in a community repository (e.g. GitHub). See the Nature Research guidelines for submitting code & software for further information.

Data

Policy information about availability of data

All manuscripts must include a data availability statement. This statement should provide the following information, where applicable:

- Accession codes, unique identifiers, or web links for publicly available datasets
- A list of figures that have associated raw data
- A description of any restrictions on data availability

The data that support the findings of this study will be made available on the Open Science Framework at the following web site: 'Enhancing Eyewitness Performance by Optimizing Context' https://osf.io/5xsny/

Field-specific reporting

Please select the one below that is the best fit for your research. If you are not sure, read the appropriate sections before making your selection.

☐ Life sciences ☒ Behavioural & social sciences ☐ Ecological, evolutionary & environmental sciences

October 2018

Behavioural & social sciences study design

All studies must disclose on these points even when the disclosure is negative.

Study description	This study used a combination of perceptual scaling and analysis of receiver operating characteristics to evaluate the performance of eyewitnesses. Perceptual scaling was accomplished for a set of facial lineup images using the method of paired comparisons. This method yielded each participant's preferences among six face images, from which we derived a quantitative scale of face similarity as described in Figure 2 of the manuscript. The distributions of votes cast for each lineup face (Figs 4A and 6) were used to estimate the likelihood of correct and incorrect identifications for a given decision criterion, using the tools of signal detection analysis.
Research sample	Subjects were either (1) undergraduate students from the University of California, San Diego (UCSD), who received course credit for their participation (96%), or (2) college-age individuals from the La Jolla community, who received monetary compensation for their participation (4%). Two hundred two (202) subjects participated in the experiments (mean age 20.7 years, standard deviation 2.9 years, range 18-35 years; 67% female). Study participants included 15% white (not of hispanic origin), 65% asian/pacific islander, 13% hispanic, 3% black (not of hispanic origin), 1% native American, and 3% other. This sample is representative of the young adult population of California. All participants had normal or corrected-to-normal visual acuity. This study sample was chosen by convenience and because it is representative with regard to the risks, benefits, and purpose of the research.
Sampling strategy	Participants were sampled by convenience as described in section Recruitment. The sample size was chosen in accord with typical sample sizes used in the literature on eyewitness identification and perceptual scaling. The chosen sample size is both manageable for a two-visit in-person laboratory study and sufficiently large to enable high-confidence judgments about differences between perceptual responses to different stimuli.
Data collection	The experiment consisted of two parts. In the first part, every participant was presented with the same video recording of a mock crime: a fragment from a theatrical film. In the second part, conducted on the following day, each participant was presented with one of three lineup types described in the manuscript: simultaneous (N=33), sequential (N=37), and paired comparison (N=132). The type of lineup was assigned to participants randomly. All participants assigned to the simultaneous and sequential lineups viewed "target-present" lineups. In this condition, one of the lineup faces was that of the actor who played the role of perpetrator in the mock crime video. The other lineup faces were fillers selected based on certain attributes (race, facial hair, etc.) possessed in common with the perpetrator. Participants assigned to the paired comparison lineup viewed either this target-present condition (N=62) or a target-absent condition (N=70). This latter condition was identical to target-present, except that the perpetrator face was removed from the lineup and replaced with another face selected based on certain attributes (race, facial hair, etc.) possessed in common with the perpetrator. All visual stimuli were rendered in color on a computer monitor in a quiet light-tight behavioral testing room. Behavioral responses were executed by a cursor press. The experimenter was was aware of the hypotheses and experimental conditions being tested, but data collection was automated and the experimenter was not present in the testing room during the experiment. Subjects were isolated in the testing room during data collection and were neither aware of the hypotheses being tested nor the experimental conditions. Further detail is available in the Methods section of the manuscript.
Timing	Data were collected during the period September 2017 to September 2018, and during the period November 2019 to February 2020.
Data exclusions	Participants were recruited from either (1) the undergraduate student population of the adjacent UCSD campus (96%), or (2) the college-age community of La Jolla (4%). The only criteria for selection were normal or corrected-to-normal vision and willingness to participate in the experiment. Of the individuals who registered for the study, none were excluded from analyses.
Non-participation	The majority of our study participants were obtained through the UCSD Psychology Department subject pool, which engages undergraduate students in psychology, cognitive science and linguistics. Two participants were dropped from the study because they failed to show up for the second day of testing.
Randomization	Participants were allocated to experimental conditions at random.

Reporting for specific materials, systems and methods

We require information from authors about some types of materials, experimental systems and methods used in many studies. Here, indicate whether each material, system or method listed is relevant to your study. If you are not sure if a list item applies to your research, read the appropriate section before selecting a response.

Materials & experimental systems

n/a	Involved in the study	
☒	☐	Antibodies
☒	☐	Eukaryotic cell lines
☒	☐	Palaeontology
☒	☐	Animals and other organisms
☐	☒	Human research participants
☒	☐	Clinical data

Methods

n/a	Involved in the study	
☒	☐	ChIP-seq
☒	☐	Flow cytometry
☒	☐	MRI-based neuroimaging

Human research participants

Policy information about studies involving human research participants

Population characteristics	See Above
Recruitment	The majority (96%) of subjects were recruited using the UCSD Psychology Department subject pool, called SONA (ucsd.sona-systems.com), which engages undergraduate students in psychology, cognitive science and linguistics. (The corresponding author, Albright, is Adjunct Professor in the UCSD Department of Psychology.) Participants recruited through this system received credits for courses enrolled in through UCSD. A smaller number (4%) of corresponding college-age subjects was recruited from the La Jolla community. These participants received monetary compensation. Self-selection bias toward these particular experiments was not expected in either case, since detailed information about the experiments was not provided in the recruitment advertisement.
Ethics oversight	In accordance with ethical standards set by US laws and regulations, we protected the welfare, rights, and privacy of human subjects who participated in these experiments. Participants were informed of their rights as experimental participants and they provided written consent. The human subject protocol and consent form were reviewed and approved by the Human Subjects Institutional Review Board of the Salk Institute for Biological Studies (protocol #17-0002).

Note that full information on the approval of the study protocol must also be provided in the manuscript.
